# Long-Term Cost-Effectiveness of Transanal Irrigation in Patients with Neurogenic Bowel Dysfunction

**DOI:** 10.1371/journal.pone.0159394

**Published:** 2016-08-24

**Authors:** Anton Emmanuel, Gayathri Kumar, Peter Christensen, Stuart Mealing, Zenia M. Størling, Frederikke Andersen, Steven Kirshblum

**Affiliations:** 1 GI Physiology Unit, University College London Hospital, London, United Kingdom; 2 ICON Health Economics, Oxford, United Kingdom; 3 Pelvic Floor Unit, Aarhus University Hospital, Aarhus, Denmark; 4 Coloplast A/S, Humlebaek, Denmark; 5 Kessler Institute for Rehabilitation, Rutgers New Jersey Medical School, Newark, New Jersey, United States of America; Case Western Reserve University, UNITED STATES

## Abstract

**Background:**

People suffering from neurogenic bowel dysfunction (NBD) and an ineffective bowel regimen often suffer from fecal incontinence (FI) and related symptoms, which have a huge impact on their quality of life. In these situations, transanal irrigation (TAI) has been shown to reduce these symptoms and improve quality of life.

**Aim:**

To investigate the long-term cost-effectiveness of initiating TAI in patients with NBD who have failed standard bowel care (SBC).

**Methods:**

A deterministic Markov decision model was developed to project the lifetime health economic outcomes, including quality-adjusted life years (QALYs), episodes of FI, urinary tract infections (UTIs), and stoma surgery when initiating TAI relative to continuing SBC. A data set consisting of 227 patients with NBD due to spinal cord injury (SCI), multiple sclerosis, spina bifida and cauda equina syndrome was used in the analysis. In the model a 30-year old individual with SCI was used as a base-case. A probabilistic sensitivity analysis was applied to evaluate the robustness of the model.

**Results:**

The model predicts that a 30-year old SCI patient with a life expectancy of 37 years initiating TAI will experience a 36% reduction in FI episodes, a 29% reduction in UTIs, a 35% reduction in likelihood of stoma surgery and a 0.4 improvement in QALYs, compared with patients continuing SBC. A lifetime cost-saving of £21,768 per patient was estimated for TAI versus continuing SBC alone.

**Conclusion:**

TAI is a cost-saving treatment strategy reducing risk of stoma surgery, UTIs, episodes of FI and improving QALYs for NBD patients who have failed SBC.

## Introduction

Damage to the central nervous system has a profound impact on the function of the large bowel and anorectum. Spinal cord injury (SCI), multiple sclerosis (MS), spina bifida (SB), and cauda equina syndrome (CE) are the most common cause of neurogenic bowel dysfunction (NBD), of which constipation and fecal incontinence (FI) are the most frequently reported consequences. NBD affects around 80% of SCI patients [1], and 34% of SB patients [2]. Furthermore, 68% of a MS population has been shown to suffer from constipation and/or FI [3]. Appropriate pharmacological management of bowel dysfunction can be challenging in that laxatives used to treat constipation or antidiarrheal drugs to treat FI carry the risk of incontinence due to loose stool or constipation, respectively. Nevertheless, without intervention, people with NBD suffer from reduced quality of life including loss of independence and control, feelings of embarrassment, anxiety and depression as well as social isolation and loss of sexual relationships [4,5].

Neurogenic bowel is also the source of considerable morbidity including urinary tract infections (UTIs), hemorrhoids, abdominal pain, rectal bleeding and prolapse, anal fissure, and autonomic dysreflexia [6–8]. These may result in additional hospital admissions. In order to regain control and optimize comfort, safety and privacy, it is critical to establish a bowel management program that is effective, timely, and sustainable with minimum physical and pharmacological interventions used [9]. A stepped pyramidal approach to treatment of bowel dysfunction has been proposed [10]. Conservative treatment options such as adjustment of diet/fluid intake, general lifestyle alteration, oral medications including stool softeners and laxatives, digital stimulation, suppositories and biofeedback constitute the base layer of the pyramid and are referred to as standard bowel care (SBC). If this is unsuccessful, the next step is to consider a minimally invasive treatment option, transanal irrigation (TAI). The upper layers of the pyramid comprise the more invasive treatment options such as nerve stimulation implants and surgical colonic irrigation. For patients refractory to these treatments, creation of a stoma may be needed [10].

TAI of the rectum and colon is intended to assist the evacuation of feces from the bowel by introducing water via the anus, and the method has been demonstrated superior to SBC [11]. Use of TAI reduces episodes of FI [12], improves quality of life [13], reduces time spent on bowel management compared with previous methods [12,13] and is well tolerated by patients [14,15]. Furthermore, TAI has been shown to result in a lower total cost to society than standard bowel management, based on efficacy outcomes from a randomized controlled trial in an SCI population [12]. However, to date no studies have considered the cost-effectiveness, including prospectively collected health-related quality of life (HRQoL) data, for long-term use of TAI in the NBD population. Hence, the aim of the current study was to investigate the long-term cost-effectiveness of the Peristeen TAI system (Coloplast A/S, Humlebaek, Denmark) in patients with NBD and an ineffective SBC regimen.

## Materials and Methods

### Ethic statement

Data used in this study was collected as approved by The National Hospital for Neurology and Neurosurgery & Institute of Neurology Joint Research Ethics Committee, reference number 07/Q0512/34. The data was anonymized and de-identified prior to the use in this analysis, hence a written informed consent was not obtained for the use in this particular setup that is based on treatment algorithms and cost estimations.

### Design of decision model

**Fig 1:**
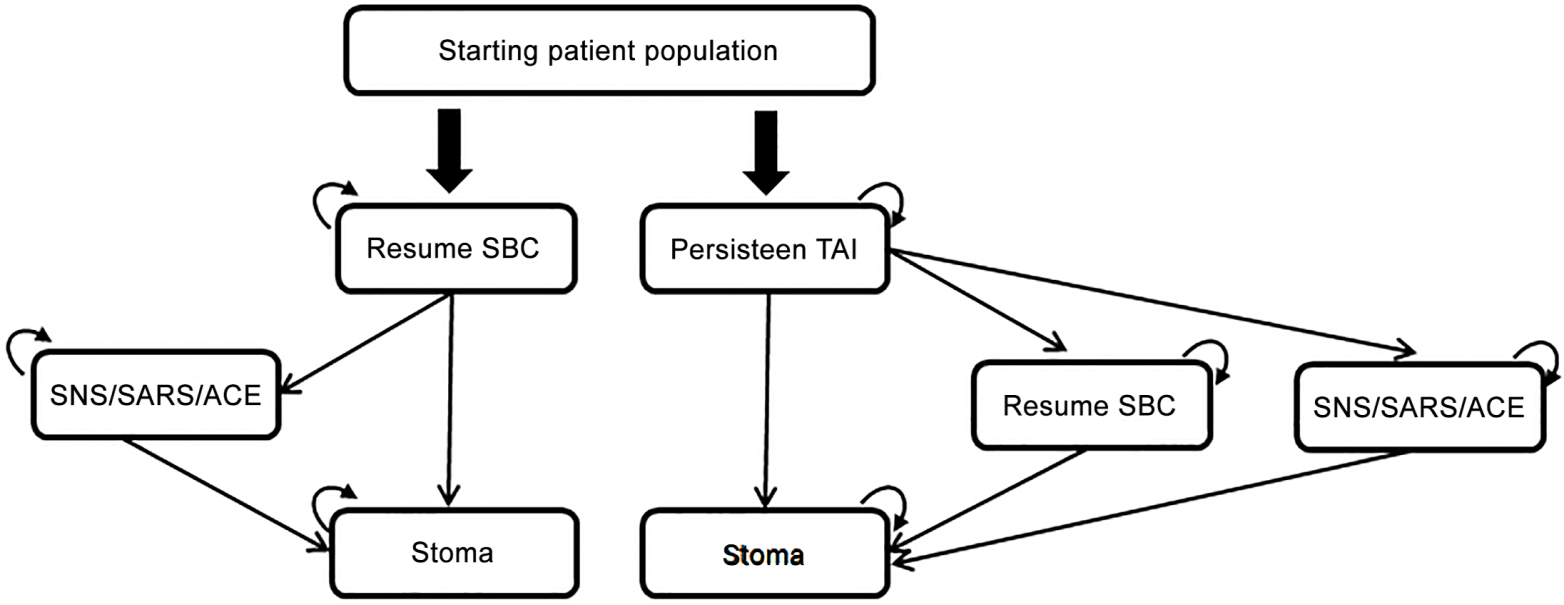
Data and Markov model overview. The boxes represent the health states that a neurogenic patient can transition between after having failed standard bowel care (SBC) before and after 2007. SBC alone. After having failed SBC >6 months, a patient can either a) Resume SBC, b) Progress to SNS/SARS/ACE or d) Progress to stoma (absorbing state). TAI in combination with SBC. After having failed SBC >6 months, a patient can either a) Initiate Peristeen TAI, b) Resume SBC, c) Progress to SNS/SARS/ACE or d) Progress to stoma. *The model assumes that patients do not transition directly from SBC/TAI to stoma*. *Transition probabilities have been obtained for each 6-month model cycle using GoalSeek in Excel*.

Markov models are valuable when modelling disease progression over time and therefore commonly applied for economic evaluation of healthcare interventions within the chronic or long-term disease area. A Markov model assumes a finite number of health states where the patient can transition between and where the cycle length is determined by e.g. treatment failure and subsequent optimization. In the present study, a deterministic Markov model was developed in Microsoft Excel^®^ (Microsoft Corporation, Redmond, WA) to evaluate the cost-effectiveness of initiating Peristeen TAI in combination with SBC in patients who had failed SBC versus continuation of SBC alone. An overview of the Markov model is presented in Fig 1 (representing each of the two treatment options under assessment). The model structure is based on the stepped pyramidal approach for treatment of NBD and illustrates the stepwise treatment modalities the patients may receive over their life expectancy. After each cycle, patients can either a) continue on TAI, b) transition to resume SBC (TAI patients only), c) transition to surgical interventions or d) transition to stoma surgery. Surgical interventions cover sacral nerve stimulation (SNS), sacral anterior root stimulation (SARS) and antegrade continence enema (ACE). Stoma surgery was defined as the absorbing state; once entered, it was not possible to transit to any other treatment states. Death was possible from all health states. A cycle length of six months was applied to the model throughout a lifetime time horizon, corresponding to the length of time a patient had to spend on failing SBC to be assessed as eligible for TAI. The time horizon corresponded to the gender-weighted life expectancy of an SCI patient diagnosed at age 30 with paraplegia A, B or C/SCI/ D [16] and was used as a base case. The model applied a United Kingdom (UK) National Healthcare Service (NHS) perspective and discount rates for costs and benefits of 3.5% p.a. Table 1 provides an overview of the transition probabilities applied in the Markov model.

**Table 1:** Overview of transition probabilities applied in the model.

Transition to/from:				
**Peristeen TAI + SBC**				
	Peristeen TAI	Resume SBC	SNS/SARS/ACE	Stoma
Peristeen TAI	0.9810	0.0069	0.0065	0.0057
Resume SBC	0.0000	0.9692	0.0113	0.0195
SNS/SARS/ACE	0.0000	0.0000	0.9891*	0.0109*
Stoma	0.0000	0.0000	0.0000	1.0000
**SBC alone**				
	Peristeen TAI	Resume SBC	SNS/SARS/ACE	Stoma
Peristeen TAI	N/A	N/A	N/A	N/A
Resume SBC	N/A	0.9736	0.0097	0.0167
SNS/SARS/ACE	N/A	0.0000	0.9831*	0.0116*
Stoma	N/A	0.0000	0.0000	1.0000

*Transition probabilities have been obtained for each 6-month model cycle using GoalSeek in Excel. SNS: Sacral Nerve Stimulation. SARS: Sacral Anterior Root Stimulation. ACE: Antegrade Continence Enema.

### Data sources

Data was collected at three clinics in the UK: University College Hospital London, Queen Square and Royal National Orthopaedic Hospital Stanmore. Since 2006, patients with bowel dysfunction, due to a neurological disorder, and referred to one of the three clinics, were added to the database. The database comprises demographic and neurological disease details as well as bowel diary data and questionnaires. Assessment is made at first visit (baseline) at the clinic and then annually thereafter in terms of the bowel parameters. The annual assessments are made at a routine clinical face-to-face visit, supported by email reminders sent a month before. The clinician or nurse specialists may if needed follow-up by phone for any missing data. Between 350 and 390 new referral are seen per year; compliance with annual follow-up is between 89 and 93% of available (namely those alive and able to communicate) patients. TAI was introduced as a treatment option in 2007. TAI was deemed suitable for patients, who had failed SBC according to the ‘best practice’ criteria’s [10], defined as those who, after at least 6 months use of two classes of laxatives, still experienced: recurrent fecal impaction, fecal urgency or incontinence, dissatisfaction with time spent toileting, dependence on cares, significant complications of constipation (abdominal pain, bloating), or negative impact on quality of life due to bowel symptoms. The prospective real-life data set used in this analysis consisted of 227 patients with NBD and a diagnosis of SCI (n = 116), MS (n = 62), CE (n = 27), SB (n = 15) or another neurological diagnosis (See Table 2 for baseline demographics). Retrospective data from 371 NBD patients pre-TAI introduction in 2007 was used to identify the proportion of patients progressing to stoma, when not having TAI as an option in the treatment pyramid. The protocols used for NBD management followed the available standards [9,10].

**Table 2:** Baseline demographics of real-life data from 227 patients.

	All	SCI	CE	MS	SB	Other
Number of participants	(n = 227)	(n = 116)	(n = 27)	(n = 62)	(n = 15)	(n = 7)
**Male:Female**	119:108	67:49	17:10	25:37	6:9	4:3
*%*	*(52*:*48)*	*(58*:*42)*	*(63*:*37)*	*(40*:*60)*	*(40*:*60)*	*(57*:*43)*
**Mean age (years)**	44	43	50	48	24	51
(range)	(17–76)	(22–71)	(20–76)	(26–69)	(17–33)	(40–4)
**Years since diagnosis**	9	7	4	13	24	2
(range)	(1–33)	(1–19)	(1–8)	(2–27)	(17–33)	(1–16)
**Walking:wheelchair**	100:127	23:93	20:7	38:24	13:2	6:1
**Normal hand function: restricted (yes:no)**	176:51	86:30	26:1	45:17	14:1	5:2
**Carer dependence (yes:no)**	100:127	76:40	1:26	22:40	1:14	0:7
**Current bladder management**						
Botox	71	31	9	28	1	2
Mitrofanoff	41	20	0	13	6	2
Urostomy	23	13	2	4	3	1
CIC*	86	49	15	15	5	2
None	6	3	1	2	0	0

*CIC: Clean Intermittent Catheterization. SCI: Spinal Cord Injury. CE: Cauda Equina Syndrome. MS: Multiple Sclerosis. SB: Spina Bifida.

Aggregate data was collected on healthcare resource utilization and the number of patients on subsequent treatment interventions at the end of follow-up. The data set contained information on frequency of FI as well as resource utilization, including number of treated urinary tract infections (UTIs), hospital admissions, and visits to general practitioner (GP), medical specialists and dieticians. The data set, including healthcare resource utilization data, was based on self-reported recall and validated in the corresponding electronic patient journals.

The proportions of patients who had transitioned from TAI to another treatment state by the end of the follow-up period were converted to six-monthly transition probabilities [17]. The estimated transition probabilities are presented in Table 2. The ‘goal seeking’ functionality in Excel was used to estimate transition probabilities from surgical interventions (SNS/SARS/ACE) to stoma by aligning the predicted proportions of patients receiving stoma at six and seven years in the model with the observed data. No long-term studies have been undertaken that are powered to detect mortality differences. It was therefore assumed that survival did not differ between the treatment options, hence the average life expectancy of an SCI patient was applied to both treatments.

Health related quality of life data was collected at baseline and at each annual follow-up visit using the EuroQoL-5D (EQ-5D) instrument, and subsequently mapped to generate single health state utility values (HSUV) on a scale from 0 (death) to 1 (perfect health) based on the UK value set [18]. Linear regression models were used to estimate disutility for patients over time. The variables for time and treatment were not significant; therefore point estimates of HSUVs at 12 months for TAI and SBC were entered into the model. Other HSUVs and decrements for UTI and hospitalization were obtained from the literature (Table 3).

**Table 3:** Health State Utility Values (HSUV).

HRQoL parameters	Input value	Reference
TAI HSUV	0.565	IPD set*
Failed SBC HSUV	0.548	IPD set*
Surgical interventions HSUV	0.548	Assumption: same as failed SBC
Stoma HSUV	0.505	Furlan 2007 [27]
UTI decrement	-0.060	NICE 2012 [28]
Hospitalisation decrement	-0.100	Watt 2012 [29]

*Individual Patient Data (IPD) set. HSUV: Health State Utility Values. NICE: National Institute for health and Care Excellence.

### Unit costs

Drug and product costs were obtained from the NHS Electronic Drug Tariff [19] and the most recent version of the British National Formulary [20]. Staff and stoma costs were obtained from the most recent Unit Costs of Health and Social Care report [21] and NHS reference costs 2013–14 [22]. All other costs were sourced from the literature on NBD management and are presented in Table 4.

**Table 4:** Costs and resource use.

**Standard Bowel Care**	**Costs**	**Frequency**	**References**
Bulking agent: Fybogel sachet (pack of 30)	£2.29	Daily	[20]; IPD set*
Softener: docusate (pack of 100)	£6.98	Daily	[20]; IPD set*
Stimulant: bisacodyl (pack of 100)	£3.43	Daily	[20]; IPD set*
Osmotic: Movicol (pack of 50)	£11.13	Daily	[20]; IPD set*
Suppository glycerine (pack of 12)	£1.94	Every alternate day	[20]; IPD set*
Suppository bisacodyl (pack of 12)	£1.57	Every alternate day	[20]; IPD set*
Enema: Norgalax 10g	£0.66	Every alternate day	[20]; IPD set*
Anal plug (pack of 20)	£44.89	Daily and per FI episode	[19]
Incontinence pad (pack of 7)	£5.95	Daily and per FI episode	[30]
**Peristeen TAI**	**Costs**	**Frequency**	**References**
System	£74.78	Irrigation every other day; replaced every 6 months	[19], [10]
Rectal catheters	£130.33	Irrigation every other day; replaced every month	[19], [10]
Initial consultation	£142.00	One-off	Consultant visit, [21]
Follow-up phone call	£100.00	Three 15-minute calls	Nurse, day ward, [21]
**Surgical interventions**	**Costs**	**Frequency**	**References**
SNS Procedure	£9,368.00	One-off	[23], inflated
SNS Follow-up	£6,286.00	Every 7 years	[23], inflated
SARS Procedure	£7,770.00	One-off	[31] (10,500 EUR converted to GBP at 1 EUR = 0.74 GBP and inflated)
SARS Follow-up	£118.92	2-monthly consultation	Colorectal surgery outpatient attendance, [22]
ACE Procedure *(weighted average of non-elective inpatient stay of major procedures for 19 years and over*, *all CC scores)*	£3,870.33	One-off	Major large intestine procedure, [22]
ACE Follow-up	£118.92	2-monthly consultation	Colorectal surgery outpatient attendance, [22]
**Stoma surgery**	**Costs**	**Frequency**	**References**
Surgery *(weighted average of non-elective inpatient stay of very complex*, *complex and major procedures for 19 years and over*, *all CC scores)*	£7,459.76	One-off	Major large intestine procedure, [22]
Colostomy bag (pack of 30)	£87.00	2 times daily	[19]
Belt (pack of 1)	£6.78	Monthly	[19]
Skin barrier (pack of 30)	£22.24	2 times daily	[19]
Adhesive remover (pack of 30)	£14.96	2 times daily	[19]
**HCP visits and caregiver**	**Costs**	**Frequency**	**References**
Consultant	£142.00	Annually (0.88, TAI; 1.04, SBC)	[21]; IPD set*
Dietician	£37.00	Annually (0.19, TAI; 0.57, SBC)	[21]; IPD set*
General Practitioner	£234.00	Annually (2.89, TAI; 3.75, SBC)	[21]; IPD set*
Caregiver salary (time spent on NBD management)	£24.00	Daily (19 minutes for 25% of TAI patients; 26 minutes for 45% of SBC patients)	[21]; [12]; IPD set*
**Adverse events**	**Costs**	**Frequency**	**References**
Hospitalizations		Annually (0.28, TAI; 1.37 SBC)	IPD set*
Gastrointestinal infection *(weighted average of non-elective inpatient stay with multiple interventions*, *with single intervention and without intervention*, *all CC scores)*	£1,998.84		Gastrointestinal infection, [22]
Pressure ulcer management	£24,214.00		[32], inflated
Falls or other trauma *(weighted average of non-elective inpatient stay*, *all CC scores)*	£2,326.32		Falls without specific cause, [22]
Abdominal pain *(weighted average of non-elective inpatient stay with and without intervention)*	£1,432.09		Abdominal pain with and without interventions, [22]
UTI *(weighted average non-elective of inpatient stay with and without interventions*, *all CC scores)*			
UTI treatment requiring antibiotics	£167.77	Annually (0.67, TAI; 1.37 SBC)	[33]; IPD set*
Peristomal skin complications	£34.89	61% annually	[34]
Hernia complication	£3,355.69	18% every 4 months	Hernia procedure, [22]
Episodes of fecal incontinence		Monthly (1.5. TAI; 3.5 SBC)	IPD set*

*Individual Patient Data (IPD) set. SNS: Sacral Nerve Stimulation. SARS: Sacral Anterior Root Stimulation. ACE: Antegrade Continence Enema.

### Model outputs

The deterministic Markov model predicts the number of FI and UTI episodes, Quality Adjusted Life Years (QALYs) and stoma surgeries, when initiating TAI versus continuing SBC in patients with NBD. Incremental cost effectiveness ratios (ICERs) are presented by comparing the cost and consequences of the two treatment options. A cost-effectiveness plane was applied to illustrate the relation between incremental cost and QALYs gained. Results are considered cost-effective within a £20,000 to £30,000 per QALY gained range as recommended by the UK National Institute for Health and Care Excellence (NICE) [23].

### Sensitivity analysis

One-way deterministic sensitivity analyses were conducted by varying HSUVs by ±0.02 and all other input values by ±25%. A probabilistic sensitivity analysis (PSA) was conducted to capture the effect of overall parameter uncertainty on the results. In the absence of information on the dispersion of parameters, standard errors were set equal to 10% of the mean parameter estimate. A value was drawn from an appropriate distribution for each parameter (with the exception of list prices) for 1,000 simulations and the impact on the costs and QALYs was plotted on a cost-effectiveness plane. A cost-effectiveness acceptability curve (CEAC) was also generated to represent the probability of cost-effectiveness of TAI relative to failed SBC across a series of willingness to pay (WTP) thresholds.

## Results

### Base case

Based on the applied data input and model assumptions the model predicts that a patient with NBD diagnosed at age 30 using TAI will experience a 36% reduction in the number of FI episodes experienced over their lifetime (from 2,069 to 1,322 episodes). The model also predicted a 35% reduction in the frequency of stoma surgery associated with the use of TAI. Furthermore, based upon self-reported recall data, the model estimated 4.46 fewer episodes (29% reduction) of treated UTIs using TAI compared with continuing SBC alone.

Overall, these clinical benefits result in an improvement in QALYs for patients receiving TAI of 0.40 compared with patients continuing SBC. The deterministic total costs and effects, and ICERs per effect outcome, are presented in Table 5. The model estimated a lifetime total cost-savings of £21,768 per patient using TAI compared with SBC. ICERs were all dominant (offering increased benefits at lower cost) regardless of whether benefits are expressed as QALYs, episodes of FI, UTIs and stoma surgery avoided.

**Table 5:** Cost-effectiveness results summary.

Comparator	Total costs	QALYs	FI episodes	UTI	Stoma
Peristeen TAI	£148,951	11.60	1322*	11.06*	0.46*
Failed SBC	£170,719	11.20	2069*	15.52*	0.71*
Incremental	-£21,768	0.40	-746.49	-4.46	-0.25
% change	-13%	4%	-36%^†^	-29%^†^	-35%^†^
ICER	—	Dominant	Dominant	Dominant	Dominant

*Predicted absolute average event counts experienced over a patient lifetime.

^†^Relative reduction over a lifetime for treatment with Peristeen TAI compared with failed SBC.

### Sensitivity analysis

**Fig 2:**
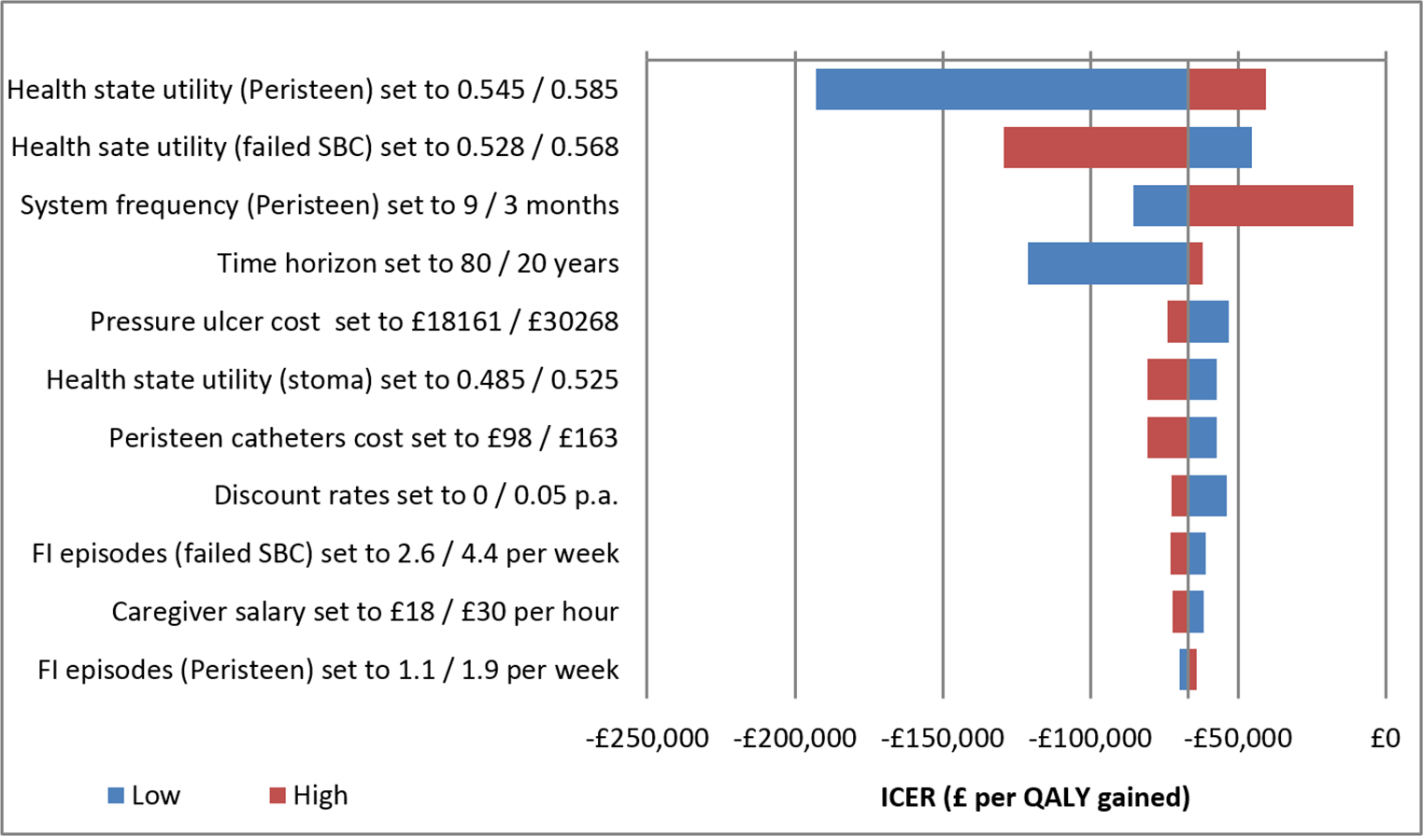
Tornado diagram.

The univariate sensitivity analyses showed that TAI was also the dominant treatment strategy associated with a cost-saving and increase in benefits, when varying key input variables ±25%. HSUV for both TAI and SBC were the most sensitive input variables. Varying the frequency with which TAI is administered per week had the third greatest impact on the results. A Tornado diagram with the most important input variables is shown in Fig 2.

**Fig 3:**
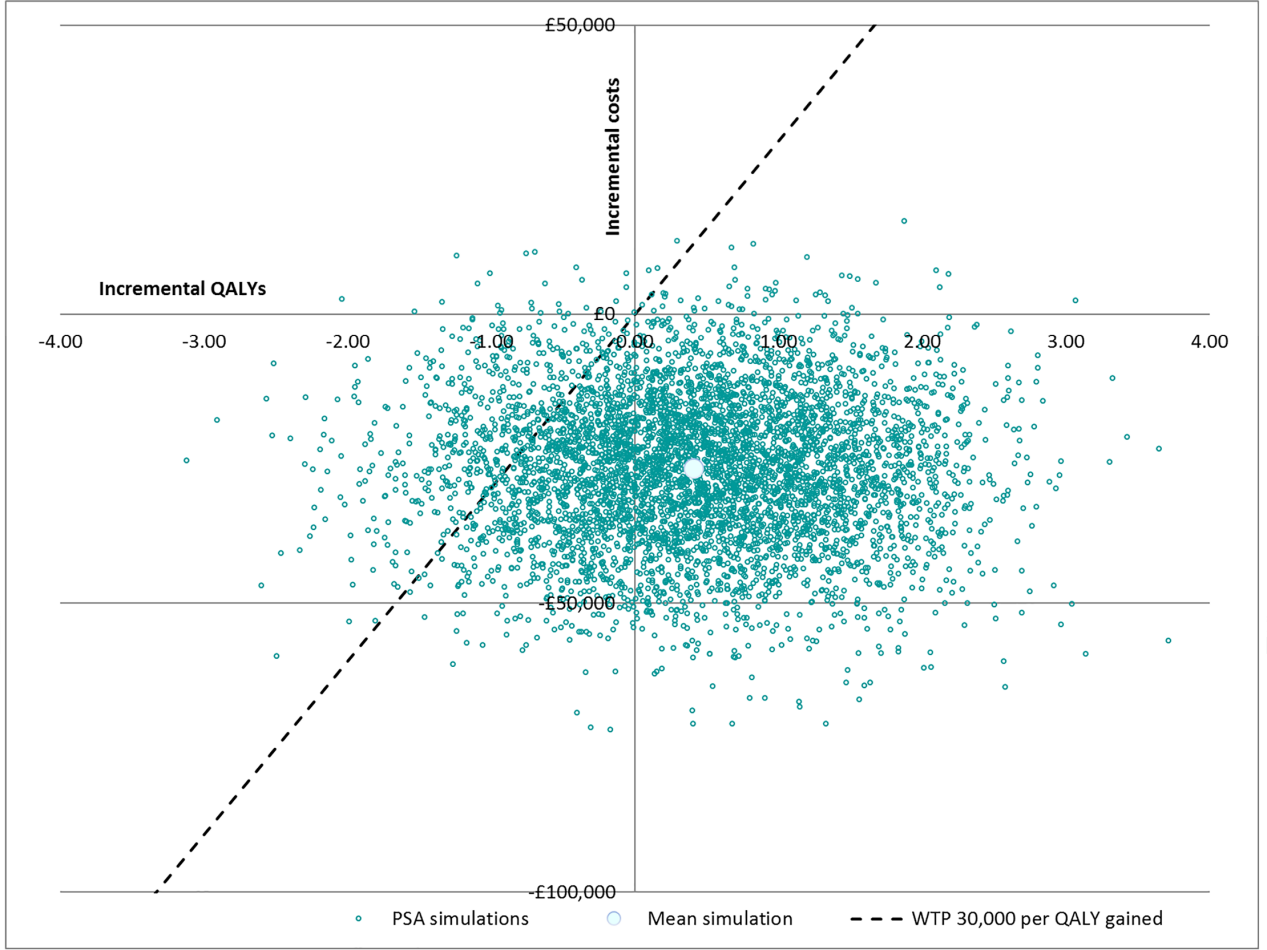
Cost-effectiveness plane. WTP: Willingness to pay: linear threshold corresponds to the WTP value used by NICE in making reimbursement decisions (£30.000 per QALY gained). Each quadrant corresponds to one incremental cost option (cost saving, not cost saving) and one incremental benefit option (more/less benefit than comparator therapy).

The incremental results of the probabilistic sensitivity analysis are presented in Fig 3. The mean incremental cost and QALYs lie in the dominant, lower right quadrant of the CE plane, indicating a positive effect on health-related quality of life (HRQOL) and that TAI is a cost saving technology. However, the dispersion of the simulations also demonstrates some uncertainty in the incremental costs and outcomes results with simulations in all four quadrants. A cost-saving is demonstrated in 96% of simulations, suggesting that while there is uncertainty about the magnitude of the cost-saving associated with TAI, it is a very robust finding that TAI is a cost-saving treatment strategy over the life expectancy of an SCI patient. The dispersion in incremental QALYs suggests that, as seen with the one-way sensitivity analysis, the model is most sensitive to HSUV inputs.

## Discussion

When new interventions and technologies are introduced to the market, they are often more effective than existing treatment strategies, but also more costly to society. To ensure optimal use of NHS resources, a cost-effectiveness threshold of £20,000-£30.000 per QALY gained has been recommended by NICE to establish which interventions represent good value for money and should be implemented (NICE 2013). In the present study, a deterministic Markov model was used to investigate the long-term cost-effectiveness of initiating TAI in patients with NBD who have failed SBC for more than six months. A unique real-life data set of NBD patients was applied in the model. The results revealed a lifetime cost saving of £21,768 and a QALY improvement of 0.40 when using TAI compared with SBC. A reduction in FI episodes, number of treatment-required UTIs, and stoma surgery were also shown. This indicates that TAI is both clinically superior for the patients and cost saving for society compared with continuing SBC alone. The practical implication is therefore that TAI should be implemented as standard treatment for patients failing SBC.

The findings confirm the results from a previously published cost-effectiveness analysis of TAI [12] showing a lower total cost to society using TAI compared with conservative bowel management. However, the 2009 study by Christensen et al [12] was based on efficacy data retrieved from a 10-week RCT where the effect of TAI was investigated in a homogeneous group of SCI patients with NBD [11]. RCTs are considered the gold standard to evaluate efficacy and safety under carefully controlled settings. This, however, can also be a drawback, as it will only reveal the efficacy in a limited study population and with a short-term time horizon. The previous cost-effectiveness evaluation of Peristeen based on the RCT study by Christensen et al [12] and the present study increases the generalizability of the findings by covering both the short-term controlled environment and the long-term real-life setting with a more heterogeneous mixed NBD study population.

In the present study, EQ-5D was used to evaluate the impact on HRQoL. The EQ-5D is a generic HRQoL-instrument used and recognized by NICE and other international HTA agencies as the preferred measure of health outcomes in health economic evaluations. The estimated QALY gain of 0.40 is modest given the increased patient benefits retrieved using TAI, e.g. reduction in episodes of FI, indicating that EQ-5D may be too generic to measure improved quality of life related to bowel management for the NBD population. In the study by Christensen et al [11], three validated bowel function-scoring systems and a symptom-related quality of-life questionnaire were used to evaluate severity of symptoms and all four showed significant improvement after 10 weeks usage of TAI. NBD bowel scores were also collected for the real-life data set used in the present study, although not included in the model. At baseline 71% of patients had an NBD score above 14, indicating a severe level of bowel dysfunction [24,25]. At the one-year follow-up after initiating TAI 32% of the patients had an NBD score above 14, representing a 55% reduction. One explanation to the small observed QALY-improvement could therefore be that EQ-5D, because of its generic form, does not capture the full HRQoL effects of TAI in the NBD population. In contrast, Liu et al [26] found a significant correlation between the severity of NBD and HRQL using the generic, but more detailed SF-36 outcome measure, which supports the argument that EQ-5D might be insensitive in this population. This discrepancy indicates that important health benefits risk being overlooked and the topic warrants further research, for example by also analyzing the EQ-5D VAS scores or by including different outcome measures.

Transition states and the majority of effect input in the present model were based on prospective real-life data collected in the UK. Transferability of the model to a setting outside of the UK would require that the analysis consider clinical practice variations, e.g. that some clinics may favor stoma surgery, whereas others will continue insufficient bowel management solutions to avoid surgery.

The estimated reduced numbers of UTIs seems low compared with results from Christensen et al [11]. However, in the present study, the frequency of UTI was based on self-reported number of treatment required UTIs, which introduces a risk of recall bias. Also, this cohort included a significant proportion with advanced bladder management strategies and less than 40% used intermittent self-catheterization, which could also influence the number of reported UTIs.

Living with a chronic condition such as NBD and the associated risks of bowel leakage or constipation have a large impact on the quality of life [5]. Finding the right bowel management solution is important both for the individual and for society. In the treatment of chronic diseases, it is important to assess the long-term effects and costs. Using costs and real-life data from a single country, this cost-effectiveness analysis has demonstrated that it is possible to model a chronic disease course along with lifetime costs when adopting a limited set of conservative assumptions.

## Conclusion

This study demonstrates that TAI is a cost saving treatment strategy for patients with NBD who have failed SBC for more than six months. In addition, TAI improves QALYs and reduces episodes of FI, UTIs and the risk of stoma surgery. The time horizon of the study was based on a lifetime perspective and a healthcare provider perspective. The sensitivity analysis showed that the results were robust to changes in key variables. TAI improves health and health related quality of life for people suffering from NBD while also saving costs to society and should therefore be implemented as a standard treatment for this patient group, when treatment with SBC is insufficient.
